# The impact of radiotherapy late effects on quality of life in gynaecological cancer patients

**DOI:** 10.1038/sj.bjc.6605050

**Published:** 2009-04-21

**Authors:** C L Barker, J A Routledge, D J J Farnell, R Swindell, S E Davidson

**Affiliations:** 1 Department of Clinical Oncology, the Christie NHS Foundation Trust, Manchester, UK; 2 Academic Department of Radiation Oncology, University of Manchester, the Christie NHS Foundation Trust, Manchester, UK; 3 Department of Statistics, the Christie NHS Foundation Trust, Manchester, UK

**Keywords:** radiotherapy, late toxicity, quality of life, gynaecological cancer, longitudinal study

## Abstract

The aims of this study were to assess changes in quality of life (QoL) scores in relation to radical radiotherapy for gynaecological cancer (before and after treatment up to 3 years), and to identify the effect that late treatment effects have on QoL. This was a prospective study involving 225 gynaecological cancer patients. A QoL instrument (European Organisation for the Research and Treatment of Cancer QLQ-C30) and late treatment effect questionnaire (Late Effects Normal Tissues – Subjective Objective Management Analysis) were completed before and after treatment (immediately after radiotherapy, 6 weeks, 12, 24 and 36 months after treatment). Most patients had acute physical symptoms and impaired functioning immediately after treatment. Levels of fatigue and diarrhoea only returned to those at pre-treatment assessment after 6 weeks. Patients with high treatment toxicity scores had lower global QoL scores. In conclusion, treatment with radiotherapy for gynaecological cancer has a negative effect on QoL, most apparent immediately after treatment. Certain late treatment effects have a negative effect on QoL for at least 2 years after radiotherapy. These treatment effects are centred on symptoms relating to the rectum and bowel, for example, diarrhoea, tenesmus and urgency. Future research will identify specific symptoms resulting from late treatment toxicity that have the greatest effect on QoL; therefore allowing effective management plans to be developed to reduce these symptoms and improve QoL in gynaecological cancer patients.

An increase in survival rates over the last few decades (Cancer Research UK, 2006) has lead to a greater proportion of patients living with the late adverse effects of cancer treatment. The effect of these long-term problems on a patient's physical, social and psychological well-being needs to be addressed in oncology research and practice.

Around one in ten women in the UK living with a diagnosis of cancer will have a gynaecological malignancy (Forman *et al*, 2003). There are particular challenges in addressing the long-term needs of gynaecological cancer survivors. Patients report more gastrointestinal symptoms and sexual dysfunction than women in the general population, even several years after therapy (Bergmark *et al*, 1999; Bye *et al*, 2000; Klee and Machin, 2001). In addition, treatment is associated with a loss of fertility and the use of long-term hormone replacement therapy to control menopausal symptoms.

Surgery, radiotherapy and chemotherapy are effective treatment options for patients with gynaecological cancers, and these are increasingly being used in combination to improve survival (Green *et al*, 2001; Ryn *et al*, 2005). The use of multimodal treatment regimes is associated with a rise in the incidence of late treatment effects seen within the pelvis (Landoni *et al*, 1997; Creutzberg *et al*, 2000). These are defined as toxicity present at least 3 months after the completion of radiotherapy (Maher and Denton, 2008). Despite an increasing trend in using multimodal treatment regimes in oncology practice, there are a paucity of data on long-term treatment effects (Kirwan *et al*, 2003) and their subsequent effect on quality of life (QoL) in gynaecological cancer patients.

Health-related QoL is a subjective patient estimation regarding the effect of disease and treatment variables on the outcomes of emotional, social, physical and functioning well-being. There are many interactions between symptoms, functional status and overall QoL (Osoba, 2007), and it cannot be linked in a straightforward way to a particular degree of organ or tissue damage (Maher and Denton). There are several studies in the literature that explain the physical, psychological and social effects of gynaecological cancer and its treatment (presented in Table 1). Many of these studies are prospective and they describe the initial decrements in QoL immediately after therapy. However, the length of time before complete recovery is unclear, with the longest prospective study showing that QoL never reached that of healthy controls after a 24-month period (Klee *et al*, 2000a). Crucially these studies do not take into account the symptoms experienced from late treatment effects, which typically manifest around 2–3 years after treatment (Denton *et al*, 2000), and their consequent effect on QoL.

It is important to measure QoL and late treatment effects accurately in order to identify, and therefore effectively manage those symptoms causing the greatest distress after treatment. Instruments measuring QoL should be reliable, valid and responsive to changes over time (Cella *et al*, 2002). They should also take a ‘multidimensional’ approach that involves assessing physical, functional, social and emotional well-being. Many of the more commonly used and well-validated questionnaires include domains in these areas. In addition, they have site-specific modules that can be used wherever relevant.

In contrast, there is no general consensus on the best way to quantify normal tissue damage and to record treatment-related effects (Bentzen *et al*, 2003). Reports of the frequency of late treatment effects from pelvic radiotherapy vary, and this may reflect the different techniques used to score treatment-related toxicity (Hoeller *et al*, 2003). This variation may also be because of a lack of documentation of these effects, especially those deemed less serious by health professionals (Davidson *et al*, 2003). The Late Effects Normal Tissues (LENT) – Subjective Objective Management Analysis (SOMA) scoring system (LENT SOMA tables, 1995) addresses both the patient's view on their symptoms and a clinician's opinion on morbidity, and therefore provides a comprehensive measure of late treatment effects. These LENT SOMA items have subsequently been incorporated into the National Cancer Institute's (NCI) Common Terminology Criteria for Adverse Events (CTCAE), which includes a comprehensive catalogue of potential normal tissue effects.

There were two main objectives of this QoL study. The first was to examine the QoL of women undergoing radiotherapy for treatment of gynaecological cancer (before and after treatment up to 3 years using European Organisation for the Research and Treatment of Cancer (EORTC) QLQ-C30 (Aaronson *et al*, 1993)) prospectively. The second aim was to identify the effect of late treatment effects assessed using the LENT SOMA scales on the QoL of these patients.

## Materials and methods

The gynaecological cancer patients that were approached to take part in the study (between 1998 and 2006) were only those patients that were to receive radical radiotherapy treatment (radiotherapy with curative intent). A total of 225 patients were recruited prospectively at the Christie Hospital, Manchester. Patients were asked to complete questionnaires either by interview with a research nurse or self-administered at home and returned by post.

Approval for this study was obtained from the South Manchester Research Ethics Committee. Patients who developed disease recurrence or had progression of the disease were excluded from this study.

At the time of completion, patients were also asked whether they wished to discuss any concerns they may have had in a consultation with a doctor. They were also asked to document any issues not related to their cancer that may influence their QoL scores (e.g., the breakdown of a relationship may have a negative effect on QoL).

The first questionnaire was completed before the start of radiotherapy and served as a baseline assessment. Time periods after treatment were measured from the last day of treatment. The post-radiotherapy questionnaires were completed after treatment (from 9 days before end to 12 days after completion of treatment) at 6 weeks after radiotherapy (±2 weeks), and at 12 months (±4 weeks), 24 months (±4 weeks) and 36 months (±4 weeks). Any questionnaires completed outside the designated range were not included in the statistical analysis.

Sixty-one patients received radiotherapy alone for treatment of a gynaecological malignancy. Ninety-three patients received radiotherapy treatment after radical hysterectomy either because of nodal involvement on histological examination or if the surgical margins were considered unsatisfactory (when vault intra-cavity treatment was also given). Concomitant chemoradiation (without surgery) was used in the treatment of 42 patients and a combination of all three treatment modalities was used for 29 patients.

The EORTC QLQ-C30 is a cancer-specific 30-item questionnaire intended for use in clinical trials (Aaronson *et al*, 1993). It was designed to be multidimensional, self-administered and acceptable across a range of cultures. For the EORTC QLQ-C30 questionnaire, 30 question scores were transformed according to the EORTC QLQ-C30 scoring manual (Fayers *et al*, 1999).

The LENT SOMA scales were originally published in 1995 and a 37-item questionnaire was produced to contain the LENT subjective scales (Davidson *et al*, 2003). The latest version of this questionnaire can be found at the Christie Hospital website (www.christie.nhs.uk).

A programme was written to score the LENT SOMA scales according to the criteria proposed in the 1995 published tables. The questionnaires were divided into six subsites: uterus/cervix, ovary/reproductive, rectum/bowel, bladder/urethra, ureter/kidney and vagina (including questions on sexual function). Each symptom was scored with increasing severity on a scale of 0–4; with 4 being the highest intensity. An average score was calculated from the questions asked about each subscale. If >50% of the questions were missing in any one subscale or for any one person, then the average score was defined as missing and the data were not used. An overall LENT SOMA score was also formed for all the questions in the questionnaire.

### Statistical analysis

As the data were not normally distributed nonparametric statistics were used. Friedman's two-way analysis of variance was not utilised to examine the changes in scores with time, as the sample size was small at 36 months after treatment. Instead the Wilcoxon matched pairs signed rank test was used and a Bonferroni correction applied. Therefore, all changes in scores with time (e.g., from baseline assessment to 6 weeks after treatment) are reported at a reduced level of 0.01 significance (0.05 per number of tests). Mann–Whitney *U* and Kruskal–Wallis tests were used to analyse statistically significant differences between groups. Correlations between the EORTC QLQ-C30 global QoL scale and age were carried out by determining the Spearman's correlation coefficients.

## Results

### Compliance

Of the 225 patients taking part in this research, nine did not wish to continue taking part and 39 patients stopped returning questionnaires during the lifetime of the study. Patients excluded from the study included 35 patients who developed recurrence and 14 patients who died during the 3-year period. This gave a total of 176 patients, who have either completed or been withdrawn (developed recurrence or died) from this prospective study, with 49 patients still at various time points after treatment. Complete data regarding compliance were only available for 176 of the 225 patients; therefore, it was determined from this set of 176 patients that the percentage that stopped returning questionnaires (i.e., 39 patients) was equal to 27.3%. This gave an overall level of compliance of 72.7%.

Of the 225 patients taking part in this research, a total of 222 patients returned completed questionnaires at the pre-treatment assessment and 183 patients completed the questionnaires immediately after treatment. The total number of completed LENT SOMA and EORTC QLQ-C30 questionnaires returned at 6 weeks after treatment was 61, at 12 months it was 83, at 24 months it was 65 and finally at 36 months 45 patients completed the questionnaires.

The patient characteristics are summarised in Table 2.

### QoL scores over time

**Figure 1:**
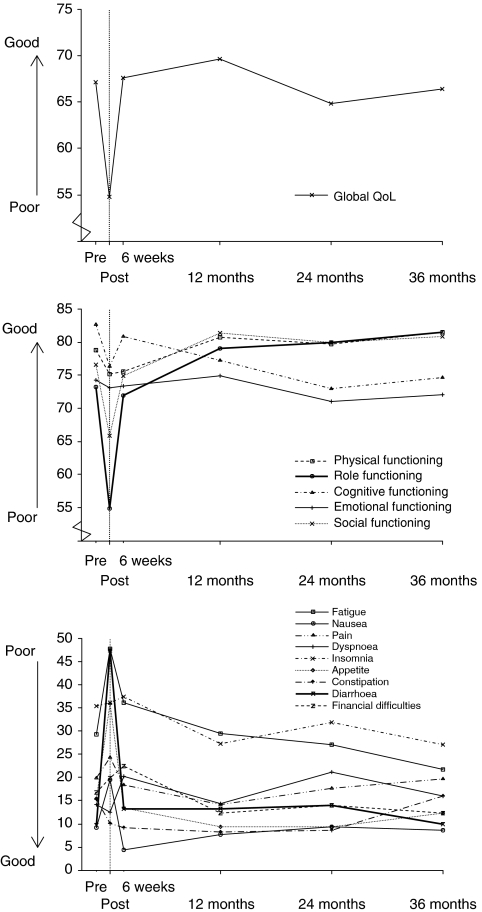
European Organisation for the Research and Treatment of Cancer QLQ-C30 subscales over time (months). Higher scores on the global quality of life (QoL) and functional scales represent better QoL, whereas higher scores on the symptom scales correspond to a higher level of symptom experience, and therefore worse QoL (range 0–100).

Figure 1 provides an illustration of QoL (EORTC QLQ-C30 subscales) throughout the course of treatment for gynaecological cancer and the 3-year follow-up. Baseline scores provided by assessments completed before the start of radiotherapy were compared with subsequent QoL results.

Table 3 shows the EORTC QLQ-C30 subscale scores (mean and median) over the study. High scores on the global QoL and functional scales represent good QoL; therefore, an increase in scores over time signifies an improvement in QoL. Immediately after treatment, scores for global QoL and physical, role, cognitive and social functioning were all decreased significantly when compared with pre-treatment levels (*P-*value <0.01).

High scores on the symptom scales of the EORTC QLQ-C30 signify a high level of symptom experience and therefore poor QoL. An increase in scores over time represents a deterioration in QoL. Immediately after treatment, symptom scores for fatigue, nausea, loss of appetite and diarrhoea were all significantly higher than at pre-treatment assessment. At 6 weeks after radiotherapy, symptom scores for fatigue and diarrhoea were still significantly increased when compared with pre-treatment assessment.

At 12 months after the end of radiotherapy, no scores for any of the EORTC QLQ-C30 subscales were significantly different from those taken at pre-treatment assessment; therefore, by 1 year all subscale scores had returned to baseline levels.

### Treatment-related toxicity scores over time

**Figure 2:**
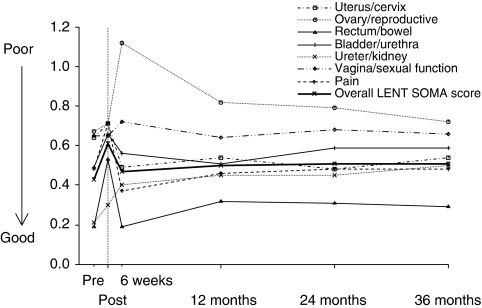
Late Effects Normal Tissues (LENT) – Subjective Objective Management Analysis (SOMA) subscales over time (months). Higher scores on the LENT SOMA scales indicate a higher level of symptom experience from treatment-related toxicity (range 0–4).

Figure 2 provides an illustration of treatment-related toxicity (LENT SOMA subscale scores) throughout the course of treatment for gynaecological cancer and the 3-year follow-up. Baseline scores provided by assessments completed before the start of radiotherapy were compared with subsequent treatment-related toxicity results.

Table 4 shows LENT SOMA subscales scores (mean and median) over the study. High scores on the subscales of the LENT SOMA indicate high levels of symptom experience from treatment-related toxicity. Both bowel and overall LENT SOMA scores showed significant increases from pre-treatment assessment at all time points after treatment (*P-*values <.01).

Immediately after treatment, LENT SOMA bladder/urethra and pain scores were significantly increased when compared with pre-treatment assessment. At 6 weeks after the end of radiotherapy, ovary/reproductive, ureter/kidney and vagina/sexual function scores were significantly higher. Ureter/kidney scores remained significantly increased at all time points from 6 weeks after radiotherapy. Conversely, uterus/cervix scores showed significant decreases at 24 months after treatment. Finally at 36 months after treatment, scores for bladder/urethra were significantly increased from pre-treatment assessment.

### Relationship between QoL and late treatment effects

In order to analyse the relationship between QoL and the level of symptom experience from late treatment effects, the sample was divided into two groups of patients according to their overall LENT SOMA scores. These were calculated from the LENT SOMA questionnaire completed at the same time point as the EORTC QLQ-C30. One group included those patients with an overall LENT SOMA score below the mean value (the ‘low’ LENT SOMA group), with the second group consisting of those patients with a score above the mean value (the ‘high’ LENT SOMA group).

**Figure 3:**
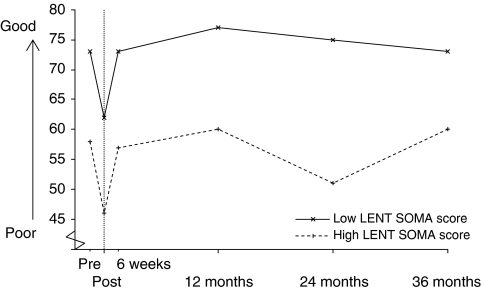
European Organisation for the Research and Treatment of Cancer QLQ-C30 global quality of life subscale over time for the ‘high’ and ‘low’ Late Effects Normal Tissues – Subjective Objective Management Analysis (LENT SOMA) score groups.

Figure 3 shows the difference in global QoL scores between the two groups over time. At all the time periods (except 36 months) there were statistically significant differences between the two groups (*P*<0.05). The clear trend was for global QoL scores to be significantly lower in those patients in the high LENT SOMA score group.

The mean rank was lower in the ‘high’ LENT SOMA group than the ‘low’ LENT SOMA group. This does show a link between high overall LENT SOMA scores and decreased QoL at this time point. This was not statistically significant however, and could be because of the small number of patients included in the analysis at this time point (*n*=27).

### Relationship between median global QoL score and disease stage, treatment modality and patient age

To analyse the different stages of disease, the sample was split into patients with earlier cancer stages (I, II) and patients with advanced disease (III, IV). Although higher stage disease was associated with a lower global QoL subscale score, the only statistically significant difference between the two groups was at the 12-month assessment (*P*=0.012).

Additionally the number of different treatments each patient received was compared with the median global QoL score at each time point. Radiotherapy, chemotherapy and surgery were each counted as distinct treatment types. There were no statistically significant differences between median global QoL scores for the number of treatment modalities received at any time point (all *P-*values >0.05).

Finally the age of the patient (calculated from the end of radiotherapy treatment) was compared with the median global QoL score at each time point. There were no significant correlations between age and median global QoL scores at any point before or after radiotherapy treatment (all *P-*values >0.05).

## Discussion

This longitudinal study was carried out to assess QoL scores over time in gynaecological patients treated with radiotherapy. With an increasing trend for using multimodal treatment regimes, and a lack of data on long-term toxicity, this research is well placed to identify both acute and late treatment effects and their associated effect on QoL scores.

The results from this study showed that patients have a high level of impairment in most of the QoL subscales immediately after treatment. These decrements are well documented in the literature with most prospective studies (Klee *et al*, 2000a, 2000b; Klee and Machin; Greimel *et al*, 2002; Distefano *et al*, 2008) supporting a known increase in physical symptoms and impaired level of functioning directly after radiotherapy. On average there was a mean change score of about 5–10 on the EORTC QLQ-C30 subscales between before and immediately after treatment. This therefore indicates a clinically meaningful change in QoL after radiotherapy treatment (King, 1996; Osoba *et al*, 1998).

Quality of life symptom scores for fatigue remained high until at least 6 weeks after radiotherapy. Fatigue is a known side effect of radiotherapy (Jereczek-Fossa *et al*, 2002). It tends to gradually increase during treatment and then decline over time, returning to pre-treatment levels 1–2 months after the end of radiotherapy. The results from this study concur with these observations. By 1 year all of the EORTC QLQ-C30 subscale scores had returned to pre-treatment levels. This result agrees with findings from a prospective study conducted by Lutgendorf *et al*, who reported an increase in QoL and mood in gynaecological cancer patients in the first year after treatment.

The LENT SOMA scores showed significant increases in treatment toxicity immediately after radiotherapy for the rectum/bowel, bladder/urethra, pain and overall LENT SOMA scales. For both the rectum/bowel and overall LENT SOMA scales, scores were significantly elevated at each time point until 3 years after radiotherapy and never reached the pre-treatment levels. These results correspond to the findings obtained by Klee *et al* who reported the trend for diarrhoea to become a chronic problem after irradiation. Bye *et al* also studied gynaecological cancer survivors 3–4 years after radiotherapy treatment and found diarrhoea was a common symptom. This study also found that scores for the bladder/urethra scale returned to those seen at pre-treatment assessment within 1 year after radiotherapy. However, there was a significant increase in the average bladder/urethra scores at 3 years after radiotherapy. These results correspond to past research on both acute and late toxicities related to radiotherapy treatment (Andreyev, 2007). A UK audit of gynaecological cancer patients treated in 1993 reported that the timing of treatment-related complications differed, with 75% of bowel toxicities occurring within the first 18 months and 73% of bladder toxicities documented between 2–3 years (Denton *et al*, 2000).

It is interesting that, although the overall LENT SOMA and rectum/bowel scores remained significantly increased for 3 years after radiotherapy treatment, scores for all the EORTC QLQ-C30 subscales returned to normal by 1 year. This may be because of a phenomenon in QoL research known as ‘response shift’ (Carver and Scheier, 2000); where patients may learn to cope with problems and the symptoms they experience, and therefore adjust their own internal values and standards.

In terms of treatment-related adverse effects, the global QoL subscale showed significant differences between the ‘low’ and ‘high’ LENT SOMA score groups for 2 years after radiotherapy. These findings imply that treatment morbidity has a negative effect on QoL scale scores for many years after treatment. In contrast, FIGO stage was shown to only have a statistically significant effect on global QoL scores at 12 months after treatment, and there was no association between either treatment modality or patient's age and global QoL scores.

As the rectum/bowel subscale scores were significantly raised at all time points after radiotherapy, it can be suggested that late treatment effects relating to this subscale have the biggest effect on QoL. These treatment effects include symptoms such as diarrhoea, tenesmus and urgency. Scores for the bladder/urethra became significantly raised at 3 years after treatment, and a longer prospective study is needed to ascertain whether late treatment effects of the bladder negatively affect QoL after this time point. Finally, earlier research has shown that sexual problems because of radiotherapy treatment are experienced by women (Burns *et al*, 2007). Although this study does not show significant effect of sexual function scores on QOL, more work is needed to assess the effect of late treatment effects on sexual function.

To our knowledge no other study has compared data collected from QoL questionnaires with site-specific scores obtained on late effects toxicity related to gynaecological cancer treatment. Therefore, no direct comparisons can be made with the earlier research. In Greimel *et al* (2008), patients with high-stage disease were associated with lower functioning scores on the EORTC QLQ-C30 subscales. The results obtained from this research are therefore indirectly in agreement with these findings, as high-stage disease is associated with increased symptom experience both before and after treatment (Routledge *et al*, 2003).

In this study, QoL and treatment-related toxicity were recorded simultaneously at pre- and post-treatment time points to assess changes over time and to determine any relationship between the two. The EORTC QLQ-C30 questionnaire used in this study was designed to be applicable to a wide range of cancer patients as a core questionnaire. There is now a specific cervical cancer module (EORTC QLQ-CX24) to assess QoL in patients with cervical cancer and an endometrial cancer module under development. These disease-specific measures have been shown to improve on the generic EORTC QLQ-C30 questionnaire and to provide a better assessment of the issues that affect the QoL of women treated exclusively for gynaecological cancers (Greimel *et al*, 2006). This study was initiated in 1998, however, before the introduction of the EORTC QLQ-CX24 in 2006. Hence, the core EORTC QLQ-C30 questionnaire was used here. In addition, the use of the LENT SOMA questionnaire to record treatment-related morbidity may be criticised as the system is not in widespread use. However, the items used in the questionnaire have been incorporated into the observer-based CTCAE toxicity scoring system, which has been proposed as the new standard for reporting adverse events in clinical trials (Chen *et al*, 2006).

Missing questionnaires are a frequent problem in QoL studies and may produce biased results. For this study the overall level of compliance was 72.2%, which is comparable with published reports for compliance rates in other prospective QoL studies (Kaasa *et al*, 1998). Finally all patients who experienced disease recurrence or progression of disease were excluded from this study, and, as such, our results may only be applicable to gynaecological patients with a generally ‘good’ physical condition and positive prognostic factors.

In conclusion, this study has shown that radical radiotherapy has a negative effect on QoL in gynaecological cancer patients. These effects are most apparent immediately after treatment. High treatment morbidity scores are associated with lower global QoL. Simultaneous use of both a QoL measure and a toxicity scoring system should be implemented in oncology trials and longitudinal studies. This will enable us in the future to identify the symptoms that cause the greatest distress to patients and have the biggest effect on QoL scores. In turn this will lead to effective management strategies that can be implemented to reduce the experience of these late treatment effects; therefore improving the QoL in gynaecological cancer patients treated with radiotherapy.

## Figures and Tables

**Table 1:** Earlier research on the QoL in gynaecological cancer patients

**Author**	**Study design**	**Cancer**	**Number**	**QoL instrument**	**Results**
Bye *et al* (2000)	Cross-sectional	Endometrial and cervical	79	EORTC QLQ-C36	Lower QoL in the areas of diarrhoea and role functioning than in normal population 3–4 years after radiotherapy. Pain and diarrhoea associated with decrease in QoL
Klee *et al* (2000a)	Prospective cohort	Cervical and vaginal	118	EORTC QLQ-C30	Overall QoL reduced compared with a control group, even at 24 months after treatment
Klee *et al* (2000b)	Prospective cohort	Cervical	118	EORTC QLQ-C30	Acute physical symptoms up to 3 months after treatment. Frequent voiding and diarrhoea may become chronic symptoms
Klee and Machin (2001)	Prospective cohort	Endometrial	49	EORTC QLQ-C30	Physical symptom scores were highest immediately after treatment. Global QoL lower than healthy controls
Leake *et al* (2001)	Cross-sectional	Gynaecological	202	FLI-C	Treatment with radiotherapy is associated with deterioration in QoL scores
Greimel *et al* (2002)	Prospective cohort	Gynaecological or breast	248	EORTC QLQ-C30	Decrease in global QoL, emotional functioning and role functioning up to 1 year after treatment
Lutgendorf *et al* (2002)	Prospective cohort	Gynaecological	98	FACT-G, POMS	Decrements in physical, functional and total well-being reported at baseline. Improvements in QoL and mood by 1 year
Frumovitz *et al* (2005)	Cross-sectional	Cervical	114	SF-12, BSI-18, A-DAS, CARES, FSFI	Patients treated with radiotherapy had a worse sexual functioning than those treated with surgery alone
Wenzel *et al* (2005)	Cross-sectional	Cervical	51	SF-36, QOL-CS, IES, GPC, SAQ, ISEL, FACT-Sp, COPE	QoL and functioning in cervical cancer survivors comparable with age-matched controls. Cancer-specific distress, spiritual well-being, maladaptive coping and reproductive concerns are predicative of individual QoL
Bradley *et al* (2006)	Cross-sectional	Cervical and endometrial	152	SF-36, FACT-G, CES-D, POMS	No significant differences in QoL or depressive symptoms between cancer survivors or healthy controls. However, cervical cancer survivors report more negative mood
Vistad *et al* (2006)	Critical review	Cervical	2041	Self-report measures	QoL in cervical cancer survivors is reduced compared with the general population after radiotherapy
Vistad *et al* (2007)	Cross-sectional	Cervical	79	FQ, HADS, SF-36, SAQ, LENT- SOMA	Almost one-third of cervical cancer survivors report chronic fatigue (CF). Those with CF had significantly lower QoL and higher levels of depression, anxiety and physical impairment
Distefano *et al* (2008)	Prospective cohort	Cervical	93	SF-36, HADS	QoL scores comparable between locally advanced cervical cancer patients receiving pre-operative chemotherapy and those with early stage disease. Poor QoL scores associated with anxiety disorders, low educational level and unemployment status
Greimel *et al* (2008)	Cross-sectional	Cervical	121	EORTC QLQ-C30, QLQ-CX24, SAQ	Patients treated with adjuvant radiotherapy are more likely to have impaired QoL than those treated with surgery or adjuvant chemotherapy

A-DAS=Abbreviated Dyadic Adjustment Scale; BSI-18=Brief Symptom Inventory 18; CARES=Cancer Rehabilitation Evaluation System; CES-D=Center for Epidemiologic Studies Depression scale; COPE=Coping Orientations to Problems Experienced Scale; EORTC=European Organisation for the Research and Treatment of Cancer; FACT-G=Functional Assessment of Cancer Therapy (General); FACT-Sp=Functional Assessment of Cancer Therapy-Spirituality Scale; FLI-C=Functional Living Index; FQ=Fatigue Questionnaire; FSFI=Female Sexual Function Index; GPC=Gynecologic Problems Checklist; HADS=Hospital and Depression Scale; IES=Impact of Event Scales; ISEL=Interpersonal Support Evaluation List; LENT SOMA=Late Effects of Normal Tissue, Subjective Objective Management Analysis; POMS=Profile of Mood States; QoL-CS=Quality of Life – Cancer Survivorship; SAQ=Sexual Activity Questionnaire; SF-12=Short Form 12; SF-36=Short Form 36.

**Table 2:** Patient characteristics

Number of patients recruited	225	
		
*Age at the end of radiotherapy treatment (years)*		
Mean	54.3	
Median	55.0	
Range	24–85	
		
*Cancer site*	*Number of patients*	*Per cent (%)*
Cervix	167	74.2
Endometrium	57	25.3
Vagina	1	0.5
		
*Stage of disease*		
I	71	31.7
II	85	37.9
III	38	17.0
IV	16	7.1
Unknown	15	6.3
		
*Treatment received*		
Radiotherapy alone	61	27.1
Radiotherapy and surgery	93	41.3
Radiotherapy and chemotherapy	42	18.7
Radiotherapy, chemotherapy and surgery	29	12.9

**Table 3:** EORTC QLQ-C30 subscale scores at each assessment

	**Pre-treatment**	**Immediately after treatment**	**6 weeks**	**12 months**	**24 months**	**36 months**
*Global QoL*						
Mean	67	55^**^	68	70	65	66
Median	75	50^**^	67	67	67	67
(LQ–UQ)	(58–83)	(33–75)	(50–67)	(67–83)	(58–92)	(50–83)
						
*EORTC QLQ-C30 functional scales*						
Physical functioning						
Mean	79	75^*^	76	81	80	81
Median	80	73^*^	80	87	93	93
LQ–UQ	(67–93)	(53–93)	(67–93)	(80–100)	(87–100)	(67–100)
Role functioning						
Mean	73	55^**^	72	79	80	81
Median	83	67^**^	67	100	100	100
LQ–UQ	(50–100)	(0–83)	(33–100)	(83–100)	(67–100)	(67–100)
Cognitive functioning						
Mean	83	76^**^	81	77	73	75
Median	100	83^**^	83	83	83	83
LQ–UQ	(67–100)	(33–83)	(83–83)	(67–83)	(67–83)	(67–100)
Emotional functioning						
Mean	74	73	73	75	71	72
Median	75	67	67	83	67	67
LQ–UQ	(67–83)	(33–75)	(42–75)	(67–83)	(58–83)	(67–100)
Social functioning						
Mean	77	66^*^	75	81	80	81
Median	83	67^*^	67	100	100	83
LQ–UQ	(67–100)	(17–100)	(17–83)	(83–100)	(67–100)	(67–100)
						
*EORTC QLQ-C30 symptom scales*						
Fatigue						
Mean	29	48^**^	36^*^	29	27	22
Median	22	33^**^	33^*^	11	11	11
LQ–UQ	(0–22)	(22–67)	(0–56)	(11–33)	(0–22)	(0–33)
Nausea						
Mean	9	20^**^	4	8	9	9
Median	0	17^**^	0	0	0	0
LQ–UQ	(0–17)	(0–33)	(0–0)	(0–17)	(0–17)	(0–17)
Pain						
Mean	20	24	18	14	18	20
Median	17	17	33	0	0	0
LQ–UQ	(0–17)	(0–67)	(0–33)	(0–17)	(0–17)	(0–17)
Dyspnoea						
Mean	14	13	20	14	21	16
Median	0	0	0	0	0	0
LQ–UQ	(0–0)	(0–0)	(0–33)	(0–33)	(0–33)	(0–33)
Insomnia						
Mean	35	36	37	27	32	27
Median	0	33	33	33	33	33
LQ–UQ	(0–0)	(0–67)	(33–67)	(0–33)	(0–33)	(0–67)
Loss of appetite						
Mean	16	36^**^	13	9	9	12
Median	0	33^**^	0	0	0	0
LQ–UQ	(0–33)	(0–83)	(0–33)	(0–0)	(0–0)	(0–0)
Constipation						
Mean	15	10	9	8	9	12
Median	0	0	0	0	0	0
LQ–UQ	(0–0)	(0–0)	(0–0)	(0–0)	(0–0)	(0–0)
Diarrhoea						
Mean	10	47^**^	13^*^	13	14	10
Median	0	67^**^	0^*^	0	0	0
LQ–UQ	(0–0)	(33–100)	(0–33)	(0–0)	(0–0)	(0–0)
Financial difficulties						
Mean	17	20	22	12	14	12
Median	0	0	0	0	0	0
LQ–UQ	(0–0)	(0–0)	(0–33)	(0–0)	(0–0)	(0–0)

EORTC=European Organisation for the Research and Treatment of Cancer; LQ–UQ=Lower quartile – upper quartile.

^*^P<0.01 *vs* pre-treatment.

^**^P<0.001 *vs* pre-treatment.

**Table 4:** LENT SOMA subscale scores at each assessment

	**Pre-treatment**	**Immediately after-treatment**	**6 weeks**	**12 months**	**24 months**	**36 months**
*EORTC QLQ-C30 symptom scales*						
Uterus/ Cervix						
Mean	0.64	0.71	0.49	0.54	0.48^*^	0.54
Median	0.75	0	0	0	0^*^	0.5
LQ–UQ	0.75–1.25	0–1.25	0–1.00	0–0.75	0–0.75	0–0.75
						
Ovary/Reproductive						
Mean	0.67	0.71	1.12^*^	0.82	0.79	0.72
Median	0	0	2^*^	0	0	0
LQ–UQ	0–0	0–1	0–2	0–1	0–2	0–1
						
Rectum/ Bowel						
Mean	0.19	0.53^**^	0.19^**^	0.32^**^	0.31^**^	0.29^**^
Median	0	0.62^**^	0.07^**^	0.21^**^	0.21^**^	0.14^**^
LQ–UQ	0–0.07	0.21–1.15	0–0.29	0.07–0.31	0.14–0.36	0–0.29
						
Bladder/ Urethra						
Mean	0.49	0.66^**^	0.56	0.52	0.59	0.59^*^
Median	0.29	0.43^**^	0.43	0.43	0.33	0.71^*^
LQ–UQ	0.14–0.43	0.14–1.00	0.14–0.57	0.14–1.00	0.14–0.71	0.14–1.14
						
Ureter/ Kidney						
Mean	0.21	0.30	0.37^*^	0.45^*^	0.45^*^	0.50^*^
Median	0	0	0^*^	1^*^	0^*^	1^*^
LQ–UQ	0–0	0–0	0–1	0–1	0–1	0–1
						
Vagina/sexual function						
Mean	0.65	0.65	0.72^*^	0.65	0.68	0.66
Median	0.57	0.57	0.56^*^	0.57	0.57	0.67
LQ–UQ	0.50–0.57	0.57–0.57	0.43–0.67	0.30–0.67	0.33–0.71	0.50–0.80
						
Pain						
Mean	0.48	0.71^**^	0.37	0.46	0.48	0.48
Median	0.25	0.5^**^	0.5	0.25	0	0.25
LQ–UQ	0–0.50	0–1.14	0–0.75	0–0.71	0–0.43	0–0.43
						
Overall LENT SOMA score						
Mean	0.43	0.61^**^	0.46^**^	0.50^**^	0.51^*^	0.51^*^
Median	0.32	0.47^**^	0.38^**^	0.5^**^	0.33^*^	0.59^*^
LQ–UQ	0.18–0.38	0.44–1.00	0.29–0.68	0.29–0.66	0.30–0.53	0.29–0.63

EORTC=European Organisation for the Research and Treatment of Cancer; LENT SOMA=Late Effects Normal Tissues (LENT) – Subjective Objective Management Analysis; LQ–UQ=Lower quartile – upper quartile.

^*^P<0.01 *vs* pre-treatment.

^**^P<0.001 *vs* pre-treatment.
